# Selective Retention of an Inactive Allele of the *DKK2* Tumor Suppressor Gene in Hepatocellular Carcinoma

**DOI:** 10.1371/journal.pgen.1006051

**Published:** 2016-05-20

**Authors:** Yung-Feng Lin, Ling-Hui Li, Chih-Hung Lin, Mei-Hua Tsou, Ming-Tai Kiffer Chuang, Keh-Ming Wu, Tsai-Lien Liao, Jian-Chiuan Li, Wei-Jie Wang, Angela Tomita, Beverly Tomita, Shiu-Feng Huang, Shih-Feng Tsai

**Affiliations:** 1 Department of Life Sciences and Institute of Genome Sciences, National Yang-Ming University, Taipei, Taiwan; 2 Institute of Molecular and Genomic Medicine, National Health Research Institutes, Zhunan, Taiwan; 3 Institute of Biomedical Sciences, Academia Sinica, Taipei, Taiwan; 4 Department of Pathology, Kaohsiung Medical University Hospital, Kaohsiung, Taiwan; 5 Department of Pathology, Koo Fundation Sun Yat-Sen Cancer Center, Taipei, Taiwan; 6 School of Biomedical Engineering, Science, and Health Systems, Drexel University, Philadelphia, Pennsylvania, United States of America; 7 VYM Genome Research Center, National Yang-Ming University, Taipei, Taiwan; St Jude Children's Research Hospital, UNITED STATES

## Abstract

In an effort to identify the functional alleles associated with hepatocellular carcinoma (HCC), we investigated 152 genes found in the 4q21-25 region that exhibited loss of heterozygosity (LOH). A total of 2,293 pairs of primers were designed for 1,449 exonic and upstream promoter regions to amplify and sequence 76.8–114 Mb on human chromosome 4. Based on the results from analyzing 12 HCC patients and 12 healthy human controls, we discovered 1,574 sequence variations. Among the 99 variants associated with HCC (*p* < 0.05), four are from the *Dickkopf 2* (*DKK*2) gene: three in the promoter region (g.-967A>T, g.-923C>A, and g.-441T>G) and one in the 5’UTR (c.550T>C). To verify the results, we expanded the subject cohort to 47 HCC cases and 88 healthy controls for conducting haplotype analysis. Eight haplotypes were detected in the non-tumor liver tissue samples, but one major haplotype (TAGC) was found in the tumor tissue samples. Using a reporter assay, this HCC-associated allele registered the lowest level of promoter activity among all the tested haplotype sequences. Retention of this allele in LOH was associated with reduced *DKK2* transcription in the HCC tumor tissues. In HuH-7 cells, DKK2 functioned in the Wnt/β-catenin signaling pathway, as an antagonist of Wnt3a, in a dose-dependent manner that inhibited Wnt3a-induced cell proliferation. Taken together, the genotyping and functional findings are consistent with the hypothesis that DKK2 is a tumor suppressor; by selectively retaining a transcriptionally inactive *DKK*2 allele, the reduction of DKK2 function results in unchecked Wnt/β-catenin signaling, contributing to HCC oncogenesis. Thus our study reveals a new mechanism through which a tumor suppressor gene in a LOH region loses its function by allelic selection.

## Author Summary

Liver cancer is one of the most lethal human cancers. Identifying functional alleles associated with liver cancer can provide new insights into the disease’s pathogenesis and help to advance the development of new therapeutic approaches. We conducted re-sequencing of the 4q21-25 region that frequently showed loss of heterozygosity (LOH) in liver cancer. Among the 99 variants associated with liver cancer, four are found within the *Dickkopf 2* (*DKK*2) gene. We conducted haplotype analysis of the *DKK2* promoter sequence and found that a transcriptionally inactive *DKK*2 allele was selectively retained in the tumor tissues. Additionally, by sequencing individual molecular clones, we detected 7-mer CCTCCCT sites within the *DKK2* promoter region that are involved in PRDM9 binding, pinpointing hotspots for recombination and genome instability. Furthermore, we demonstrated that DKK2 functioned as an antagonist within the Wnt/β-catenin signaling pathway. Our findings have led to the discovery of a new mechanism whereby a tumor suppressor gene in a LOH region loses its function by allelic selection.

## Introduction

Hepatocellular carcinoma (HCC) is the fifth most common cancer worldwide, and the third leading cause of cancer-related mortality, contributing to over 660,000 annual deaths worldwide [1, 2]. HCC exhibits a distinct geographic distribution of over 80% of HCC cases occurring in Southeast Asia and sub-Saharan Africa. It should also be noted that the incidence of HCC has recently increased significantly in the United States of America [3].

Late-stage HCC cases typically display more genetic alterations than hyperplasia or dysplasia lesions; these alterations include chromosomal instability, DNA rearrangements, DNA methylation, and DNA hypomethylation [4]. Several studies have identified recurrent chromosomal instability regions associated with HCC by comparative genomic hybridization (CGH) or loss of heterozygosity (LOH) mapping [5–10]. The chromosomal gain regions involve 1q, 5q, 6p, 8q, 10q, 11q, 17q, and 20q, while the chromosomal loss regions involve 1p, 4q, 6q, 8p, 10q, 13q, 16q, and 17p [11]. Several cancer genes have been identified and validated in these chromosomal instability regions. However, the mechanisms by which these genomic alterations at multiple chromosomal segments of potential oncogenes and tumor suppressor genes lead to hepatocarcinogenesis remain undetermined.

The Wnt/β-catenin pathway is involved in homeostasis, cell proliferation, differentiation, motility, and apoptosis [12]. Activation of the Wnt/β-catenin pathway frequently occurs in HCC [13, 14]. β-catenin overexpression and mutations related to this have been described during early-stage HCC development and HCC progression [15–17]. More β-catenin mutations are manifested in hepatitis C virus-associated HCC than in hepatitis B virus-related HCC [17–19]. It is interesting that β-catenin mutations are typically seen in HCC with a low-level genomic instability [20], indicating that the Wnt/β-catenin pathway could represent an alternative route to hepatocarcinogenesis.

Accumulation of β-catenin in the nucleus has been observed in 40% to 70% of HCC cases [10, 21]. Several secreted proteins are known to negatively regulate the Wnt/β-catenin pathway. These Wnt antagonists can be divided into two functional classes [22]. One involves the Wise, sclerostin and Dickkopf (DKK) families that bind directly to LRP5/6. The other consists of Wnt inhibitory factors and secreted frizzled-related proteins that bind directly to soluble Wnt ligands. The DKK family consists of secreted proteins that contain two cysteine-rich domains [23] and of four members (DKK1 to DKK4) that are able to inhibit the Wnt co-receptors LRP5/6 and Kreman 1/2 [24, 25]. Down-regulation of the DKK family, when observed in HCC, usually involves epigenetic inactivation either by methylation or via silencing by miRNA [22, 26].

## Results

### LOH of 4q22-25 in HCC

**Fig 1:**
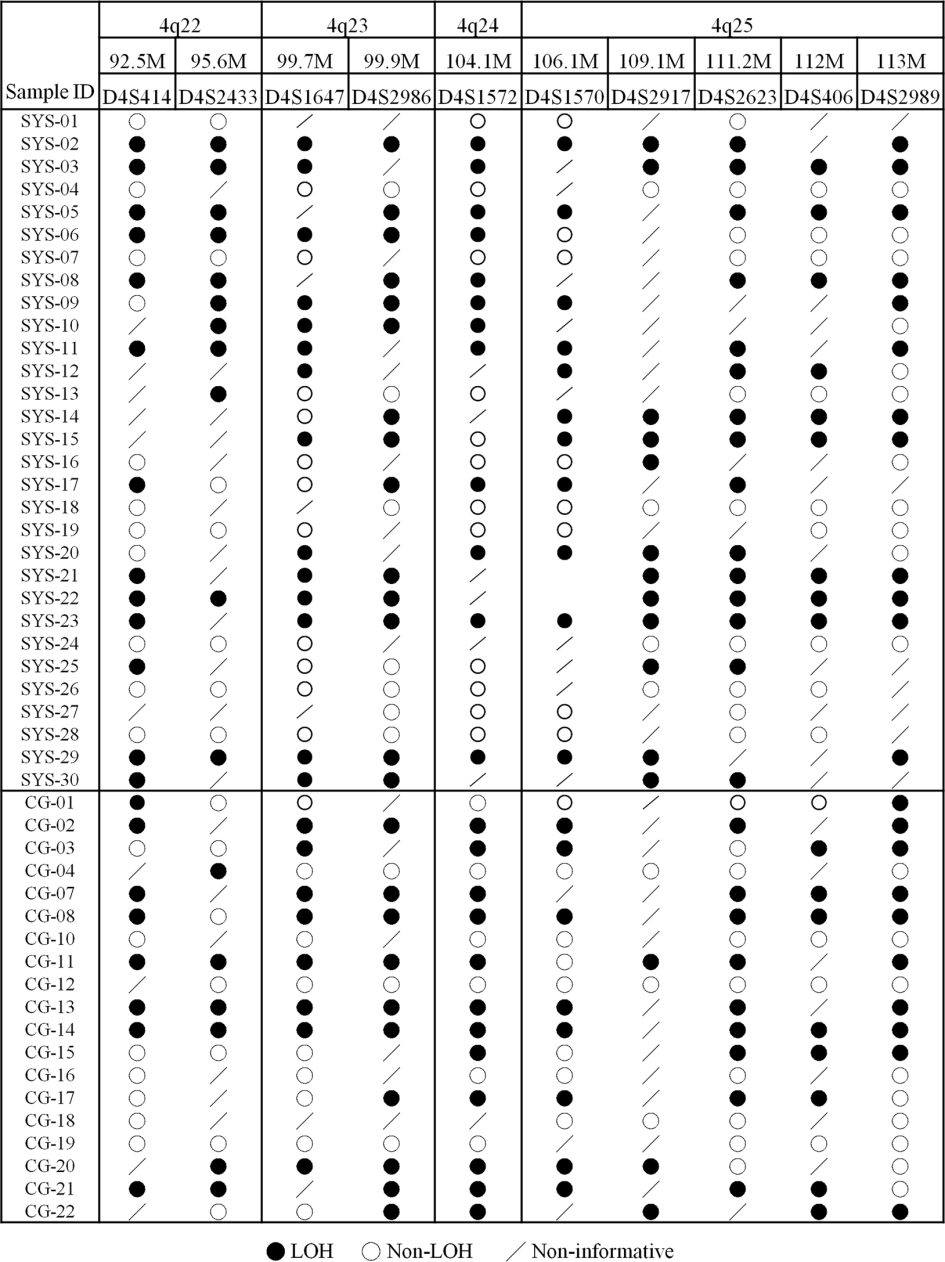
LOH of human chromosome 4q22-25 in HCC patients. The genotype status of ten STR markers from D4S414 to D4S2989 was determined for 47 cases collected from two hospitals. A solid circle indicates LOH and an open circle indicates non-LOH. A slash denotes a non-informative case.

On the basis of CGH and LOH studies, approximately 30% to 70% of HCC patients showed genetic alterations in bands 21–25 of chromosome 4q [27–29]. Chromosome 4q21-25 loss is involved in early HCC development [29]. To delineate the LOH pattern in chromosome 4q22-25, we used ten STR markers from 92.5 Mb to 117.5 Mb on human chromosome 4 to determine the minimal critical region of LOH for 47 HCC cases. As shown in Fig 1, 28 cases (59.6%) were determined to have LOH within chromosome 4q22-25 region, while the other cases were either non-informative or heterozygous. The result is consistent with the overall LOH frequencies for chromosome 4q22-25 obtained from other studies.

### Detection of variant sequences associated with HCC

According to the Knudsen’s two-hit theory [30], cancer develops when a tumor suppressor gene mutation occurs in one allele, followed by the loss of the other allele, reflecting as LOH in the genetic analysis. Thus, detection of variant sequences specifically associated with LOH in the tumor tissue is one method of identifying candidate tumor suppressor genes. We have taken a re-sequencing approach in an attempt to discover significant sequence variations in the genes on chromosome 4q21-25. A total of 2,293 pairs of primers were designed for PCR to amplify target sequences; these include 1,449 exonic and upstream promoter regions of 152 known and predicted genes that reside in the interval from 76.8 Mb to 114 Mb on human chromosome 4 (NCBI, build 33). In the pilot study using a sample panel consisting of 12 HCC patients and 12 healthy human controls, we identified a total of 1,574 sequence variations, consisting of 1,462 substitutions, 43 insertions, and 69 deletions. Among these variations, 99 sequence variations of 62 genes were found to be significantly associated with HCC (*p* < 0.05) (S1 Table).

**Fig 2:**
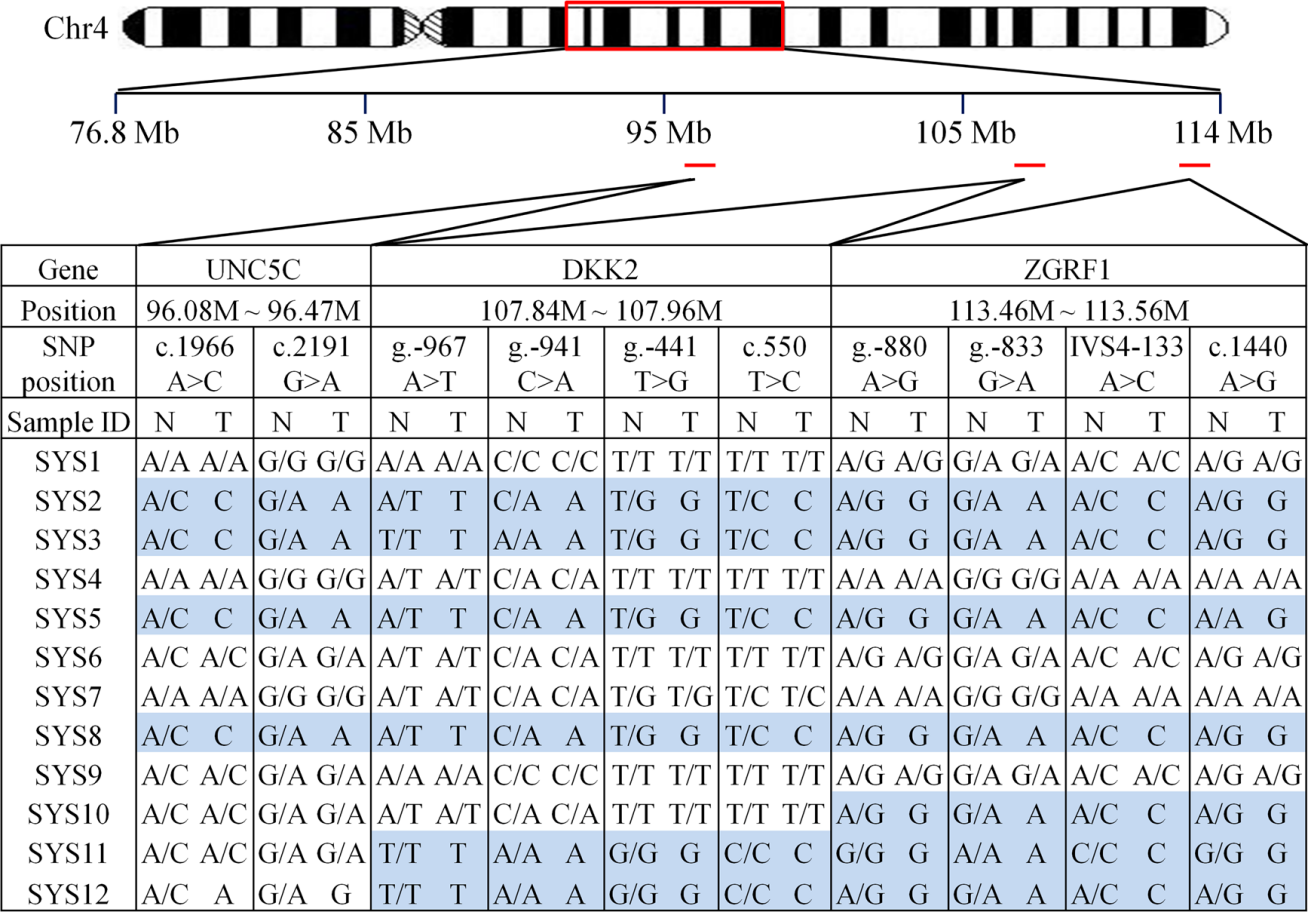
Selection of candidate genes based on allelic retention status. Genotype differences between non-tumor (designated N) and tumor (T) tissues were shown for the SNP markers of *UNC5C*, *DKK2*, and *ZGRF1* genes located in the chromosome 4 region. LOH is indicated by the shaded regions. Blue indicates the variant sequences were retained in the tumor sample, while pink denotes that the reference sequences were present in the two *UNC5C* SNPs for the sample SYS12. Note that there appear to be bias in the allelic sequences in the tumor samples.

Using allelic retention status in the HCC tumor as a criterion, three genes (*UNC5C*, *DKK2*, and *ZGRF1*) from the LOH region were evaluated for further investigation (Fig 2). *UNC5C*, which encodes ntetrin-1receptor, has been reported to function as tumor suppressor gene in human colon cancer [31, 32]. The DKK family is able to inhibit the Wnt signaling pathway in several cell types and is usually down-regulated in several different cancers [33]. *ZGRF1*, whose identity and function were not yet known at the time that we conducted the genotype analysis, is now grouped as a zinc finger gene in the database. Interestingly, 6 of the 12 cases showed LOH in the *ZGFR1* sequence and the tumors invariably retained the G-A-C-G haplotype for the four SNPs.

Of these variations that were associated with HCC, four that belong to the human *DKK*2 gene were of particular interest due to their location within the regulatory region of the gene; these consisted of three in the promoter region (g.-967A>T, g.-923C>A, and g.-441T>G) and one in the 5’UTR (c.550T>C). To further investigate the association with HCC, we increased the subject number to 47 HCC cases and 88 healthy controls to analyze these four variations. The results are summarized in Table 1. The association remained significant for *DKK*2_-967, *DKK*2_-923, and *DKK*2_+550 (*p* < 0.05). Note that the two SNPs at the promoter region (nucleotides positions -967 and -923) are in linkage disequilibrium, therefore, the allele frequency is the same between the two sites.

**Table 1:** Allele frequencies of the four variations in *DKK*2 for the HCC specimens and the healthy human control specimens.

Region	Nucleotide position	Variation	Allele frequency^a^ Control^b^ (n = 88) Patient^c^ (n = 47)	*p* value
Promoter	-967	A → T	103/73 (41.5%) 42/52 (55.3%)	0.04
Promoter	-923	C → A	103/73 (41.5%) 42/52 (55.3%)	0.04
Promoter	-441	T → G	133/43 (24.4%) 63/31 (33.0%)	0.153
5’ UTR	+550	T → C	153/23 (13.1%) 63/31 (33.0%)	2 × 10^−4^

^a^ The allele frequency of the healthy human control specimens was determined by sequencing leukocyte DNA. The allele frequency of the HCC specimens was determined by sequencing tumor tissue DNA.

^b^ The genotypes of the 88 blood samples were used to compute the haplotypes shown in Table 2.

^c^ The 47 HCC tumors were the same as those in Fig 1.

### *DKK*2 haplotypes associated with HCC

Genetic studies based on haplotypes have provided greater statistical power than those based on the underlying SNPs [34]. To investigate whether or not there were specific *DKK*2 haplotypes that are associated with HCC, we determined the haplotypes of 88 healthy controls using GENECOUNTING 2.2. A total of four haplotypes that had a probability higher than 0.02% were predicted (Table 2). Among them, two major haplotypes–haplotype 2 (ACTT) and haplotype 3 (TATT)–had a combined frequency of nearly 76% in the studied subjects; haplotype 2 was the dominant haplotype (44.9%). We also performed direct sequencing to determine the haplotypes of individually cloned genomic DNA fragments from the blood, tumor adjacent tissue, and tumor tissue of 16 HCC patients who were heterozygous for *DKK*2. Of 13 HCC cases, eight haplotypes were identified, including four recombinant haplotypes: haplotype 5 (TAGT), haplotype 6 (ACTC), haplotype 7 (TATC) and haplotype 8 (ACGT). Interestingly, these additional haplotypes were only detected in the non-neoplastic tissues but were absent from both the blood samples and tumor tissue samples (Table 3). Notably, haplotype 1 (TAGC) was the most frequently observed haplotype in the tumor tissue samples from these HCC cases, observed in 13 out of 16 samples.

**Table 2:** Predicted haplotype structure for *DKK*2.

	SNP position				
Haplotype ID	-967	-923	-441	+550	Probability^a^
haplotype 1	T	A	G	C	0.102273
haplotype 2	A	C	T	T	0.448864
haplotype 3	T	A	T	T	0.3125
haplotype 4	A	C	G	C	0.136364
haplotype 5	T	A	G	T	0
haplotype 6	A	C	T	C	0
haplotype 7	T	A	T	C	0
haplotype 8	A	C	G	T	0

^a^ Probability is based on haplotype analysis of 88 healthy human controls.

**Table 3:** Haplotypes analysis of 16 HCC cases heterozygous for *DKK*2.

	Blood			Non-neoplastic portion								Tumor part							
Sample ID	1	2	3	1	2	3	4	5	6	7	8	1	2	3	4	5	6	7	8
SYS-02	+	+	-	+	+	+	+	+	+	+	+	+	-	-	-	-	-	-	-
SYS-03	+	-	+	+	-	+	-	-	-	-	-	+	-	-	-	-	-	-	-
SYS-05	+	+	-	+	+	+	+	+	+	+	+	+	-	-	-	-	-	-	-
SYS-08	+	+	-	+	+	+	+	+	+	+	+	+	-	-	-	-	-	-	-
SYS-14	+	-	+	+	-	+	-	+	-	+	-	+	-	-	-	-	-	-	-
SYS-17	+	+	-	+	+	+	+	+	+	+	-	-	+	-	-	-	-	-	-
SYS-22	+	+	-	+	+	+	+	+	+	+	+	-	+	-	-	-	-	-	-
SYS-29	+	+	-	+	+	+	+	+	+	+	+	+	-	-	-	-	-	-	-
CG-08	nd	nd	nd	+	+	+	+	+	+	-	+	+	-	-	-	-	-	-	-
CG-10	nd	nd	nd	+	-	+	-	+	-	-	-	+	-	-	-	-	-	-	-
CG-11	nd	nd	nd	+	-	+	-	+	-	+	-	+	-	-	-	-	-	-	-
CG-14	nd	nd	nd	+	+	-	-	-	-	-	-	-	+	-	-	-	-	-	-
CG-17	nd	nd	nd	+	-	+	-	+	-	+	-	+	-	-	-	-	-	-	-
CG-19	nd	nd	nd	+	+	-	-	-	-	-	-	+	-	-	-	-	-	-	-
CG-20	nd	nd	nd	+	-	+	-	+	-	+	-	+	-	-	-	-	-	-	-
CG-22	nd	nd	nd	+	-	+	-	+	-	+	-	+	-	-	-	-	-	-	-

nd: not determined

When we compared the haplotypes of blood, tumor adjacent tissue and tumor tissue from the same patients, we unexpectedly found that there were more than two haplotypes in the tumor adjacent tissues. However, there was only one major allele, haplotype 1, retained in the tumor tissue. These results indicated that there had been frequent recombination events affecting *DKK*2 during HCC tumorigenesis and that the *DKK*2 haplotype 1 had been selectively retained in the tumors.

### Transcriptional activity of the HCC-associated *DKK*2 haplotype

**Fig 3:**
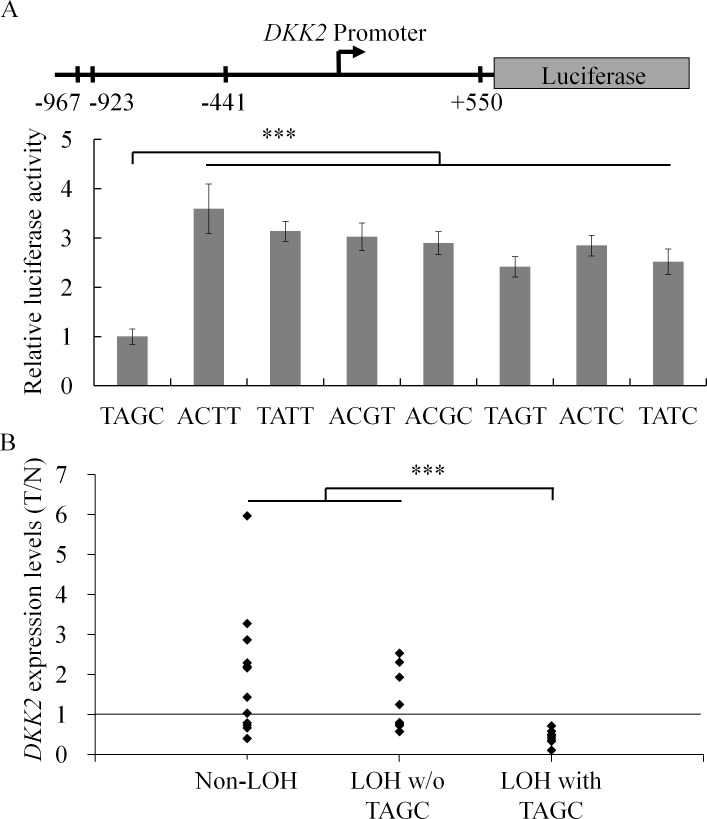
*DKK2* expression is down-regulated in tumor tissues with chromosome 4q24-25 LOH and *DKK2* TAGC haplotype. (A) Luciferase activity of the various *DKK2* haplotypes in HuH-7 cells. A reporter assay was performed to test the sequences that cover the *DKK2* promoter region from 1,135 bp upstream to 667 bp downstream of the transcription initiation site. The coding sequence of the luciferase was fused to the *DKK*2 promoter and 5’UTR sequence. The promoter function of each *DKK*2 haplotype sequence was assessed using luciferase activity, and the data obtained was normalized against the expression level of the cotransfected β-galasctosidase gene. The position of the four SNPs (at -967, -923, -441, and +550) is shown relative to the transcription initiation site. The promoter activity of the TAGC haplotype was significantly reduced relative to the other haplotypes. No significant effect on cell viability post transfection was observed for the reporter plasmids containing different *DKK2* haplotypes. (B) Relative *DKK2* mRNA expression levels of the tumor (T) tissues and the non-tumor (N) counterparts (n = 30) were assayed by RT-qPCR and classified into three categories determined by the presence of chromosome 4q24-25 LOH and/or *DKK2* TAGC haplotype. The group with chromosome 4q24-25 LOH and *DKK2* TAGC haplotype (LOH with TAGC, n = 8) showed significantly lower *DKK2* expression in tumor tissue than the other two groups: without chromosome 4q24-25 LOH (non-LOH, n = 13) and with chromosome 4q24-25 LOH but no *DKK2* TAGC haplotype (LOH w/o TAGC, n = 9). *** *p* < 0.001

Three of the four identified *DKK*2 SNPs were located in the promoter region of this gene, while the fourth was in the 5’UTR. We speculated that the various *DKK*2 haplotypes might show differences in transcriptional activity. To address this issue, we measured the reporter activity of a luciferase gene that was driven by the promoter sequences of the *DKK*2 haplotype alleles. A promoterless construct was used as a negative control, and the transcriptional activity was normalized against the transfection efficiency determined by β-galactosidase activity. Haplotype 2 (ACTT), which was found most frequently in the healthy controls, drove the expression of luciferase at a rate that was 10 fold higher than that of the promoterless construct (Fig 3A). Similarly, haplotype 3 (TATT), which is referred to as wild type in the NCBI public database, drove the expression of luciferase at a rate 8 fold higher than the reference level. In contrast, haplotype 1 (TAGC) was found most frequently in the tumor samples and showed significantly lower transcriptional activity when compared to the other seven haplotypes (*p* < 0.001). To demonstrate that *DKK2* haplotypes effect *DKK2* expression and to confirm observations from *in vitro* studies, relative *DKK2* expression levels between the tumor tissues and the non-tumor counterparts from 30 pairs HCC samples were analyzed by reverse transcription quantitative PCR (RT-qPCR). The relative expression levels were classified into three categories, determined by the presence of chromosome 4q24-25 LOH and/or *DKK2* TAGC haplotype (Fig 3B). The difference between the two groups, non-LOH and LOH without TAGC, was not significant (*p* = 0.229). However, the cohort with both chromosome 4q24-25 LOH and *DKK2* TAGC haplotype showed significantly lower *DKK2* expression levels than the other two cohorts: without chromosome 4q24-25 LOH (*p* < 0.001) and with chromosome 4q24-25 LOH but no *DKK2* TAGC haplotype (*p* < 0.001). Taken together with the genotyping data, our results indicate that this transcriptionally inactive *DKK*2 allele was being selectively retained in the tumor when heterozygous HCC patients exhibited a LOH during tumorigenesis.

### DKK2 functions as tumor suppressor

**Fig 4:**
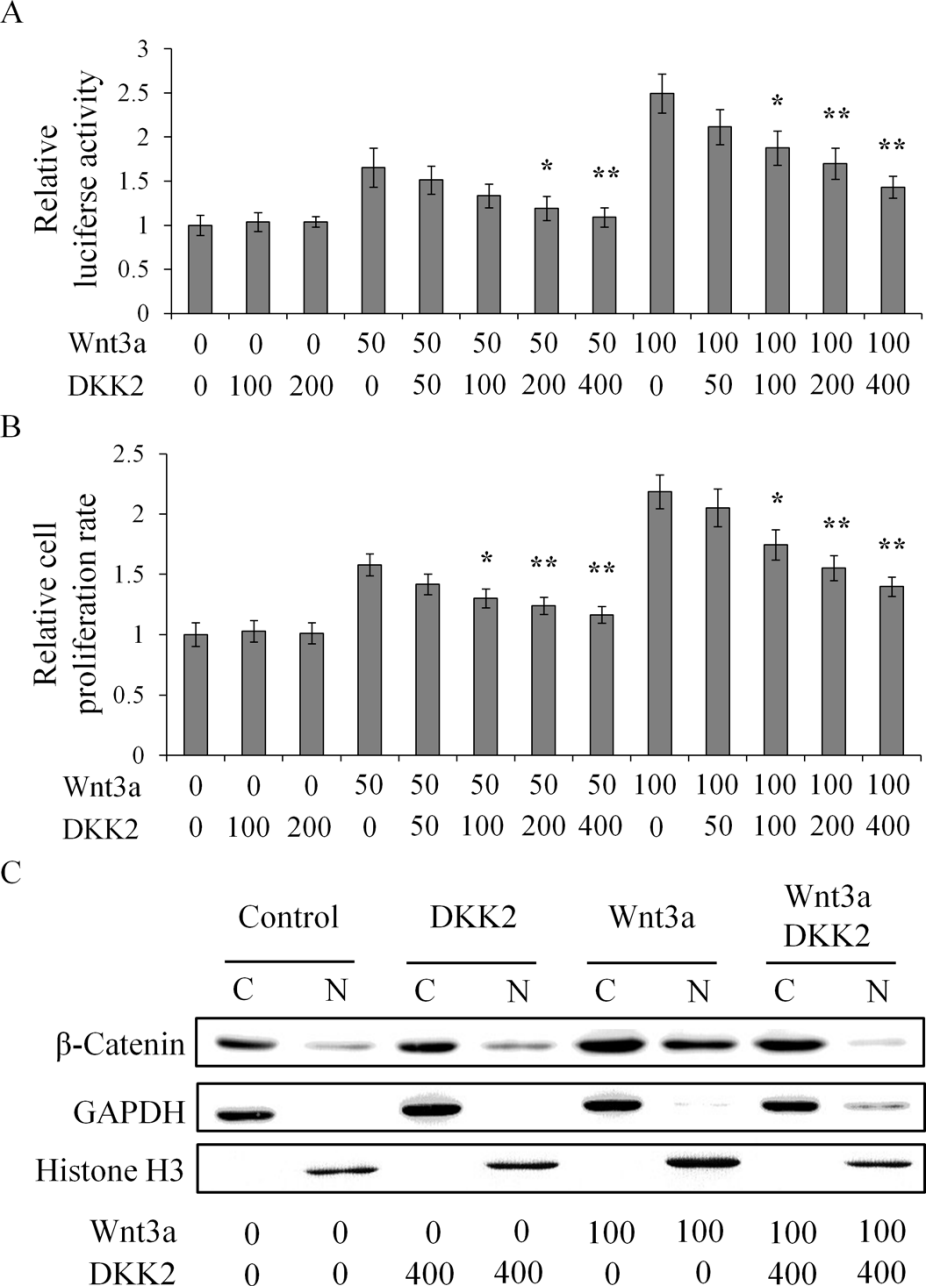
DKK2 inhibits Wnt3a-induced Wnt/β-catenin signaling. HuH-7 cells were transiently transfected with a TCF reporter plasmid and were then incubated with various concentrations of Wnt3a and/or DKK2 (ng/ml). The presence of the Wnt3a ligands activated the Wnt signaling pathway in a dose-dependent manner, as measured by elevation in the luciferase reporter activity (A). Consistently, cell proliferation increased in HuH-7 cells when they were cultured in the presence of Wnt3a ligands (B). Nuclear translocation of β-catenin was analyzed in (C), and GAPDH and Histone H3 were used to indicate cytoplasmic or nuclear fractions, respectively. Note that DKK2 addition abrogated the effects of Wnt3a significantly. * *p* < 0.05, ** *p* < 0.01 when compared to DKK2 stimulation in their respective Wnt3a levels.

To investigate the function of DKK2 as part of the Wnt/β-catenin signaling pathway in hepatocytes, we incubated HuH-7 cells that had been transiently transfected with the TCF reporter plasmid with variable amounts of recombinant Wnt3a and DKK2. The plasmid contains multiple TCF binding sites upstream of the promoter, and the luciferase activity within the cells reflected the β-catenin concentration in the nucleus [35]. As shown in Fig 4A, luciferase gene expression was correlated with Wnt3a concentration in a dose-dependent manner (*p* < 0.05). With Wnt3a stimulation, there was significant association between the luciferase activity and DKK2 concentrations above 200 ng/ml (*p* < 0.05) and DKK2 down-regulated Wnt3a-enhanced luciferase gene expression in a dose-dependent manner (*p* < 0.05). This effect was paralleled between the luciferase assay and the cell proliferation assay (Fig 4A and 4B). Consistently, by abrogating the Wnt and receptor interaction at the cell surface, DKK2 inhibited β-catenin translocation from the cytosol to the nucleus (Fig 4C). The data confirmed that signaling molecules of the Wnt/β-catenin pathway are involved in oncogenesis by controling cell proliferation [36]. Thus, the results of our DKK2 functional studies are consistent with previous reports whereby members of the DKK family are able to play a role in development and disease by modulating the Wnt/β-catenin pathway [22].

### Genetic alteration at the *DKK2* locus

**Fig 5:**
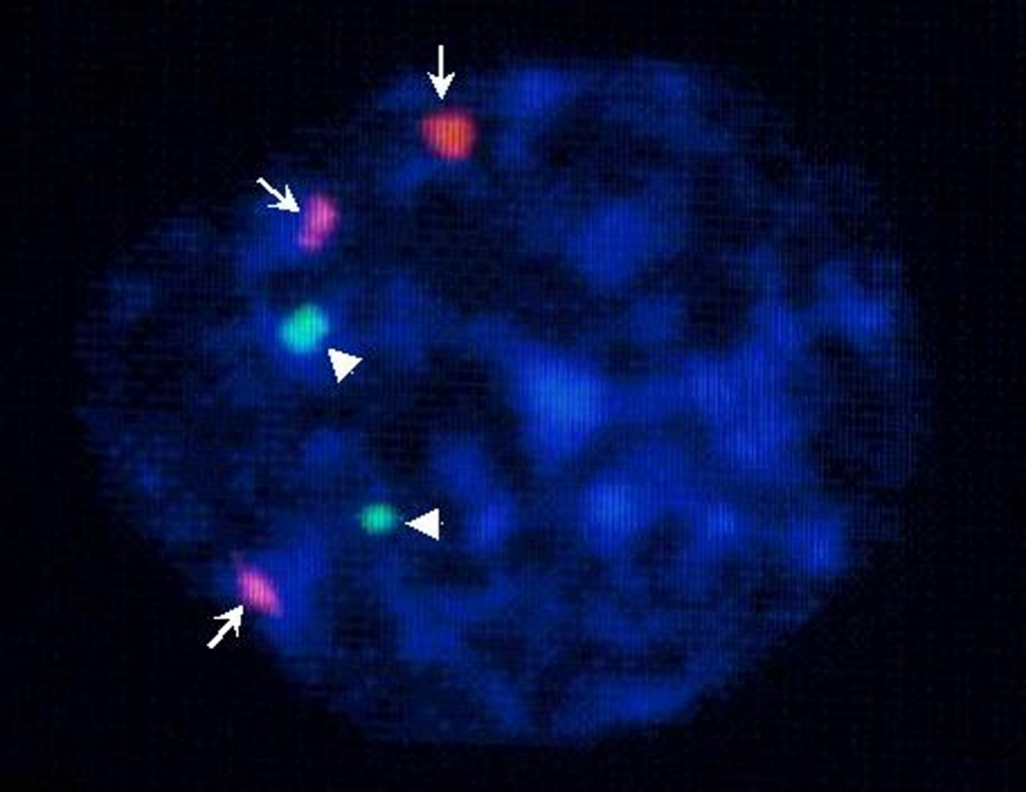
Polysomy of chromosome 4 and *DKK*2 deletion in a HCC case. The arrow indicates hybridization signals from a centromeric probe for chromosome 4. The arrowhead indicates hybridization signals from a 4q21 YAC probe. Note that there are three signals from the centromere but only two for 4q21.

**Fig 6:**
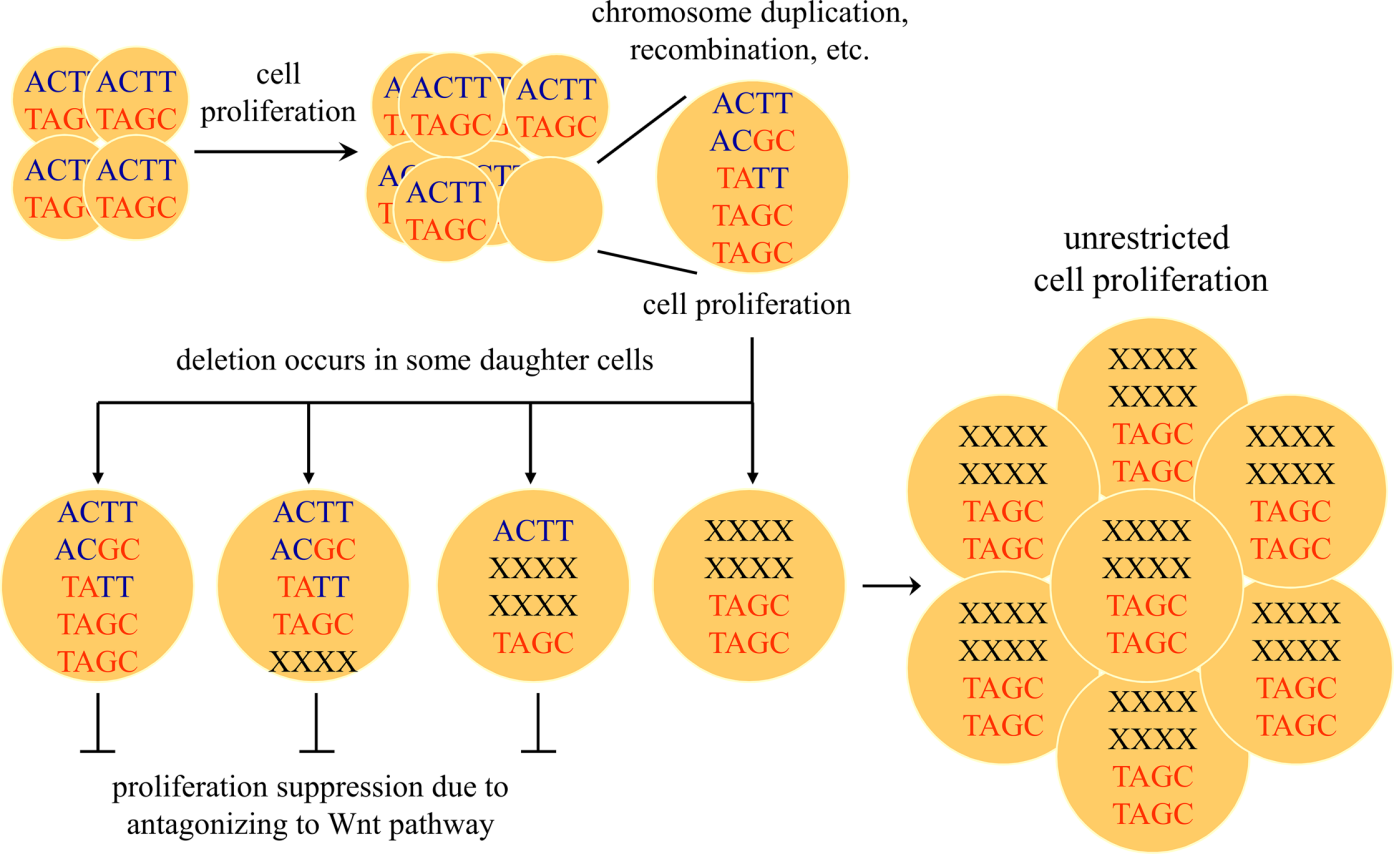
A model of HCC tumorigenesis showing the selective retention of the low transcriptional activity of *DKK*2 haplotype 1 (TAGC) allele. The cell proliferation rate is elevated in the pre-cancerous liver tissues, and a DNA break occurs near the recombination hotspots in the *DKK*2 promoter region, which leads to loss of the *DKK*2 alleles. In this scheme, those cells with low *DKK*2 transcriptional activity are selected for clonal amplification as a result of unchecked Wnt signaling.

To address possible mechanisms of LOH for the *DKK*2 gene, we analyzed the cytogenetic changes in eight HCC cases that were heterozygous for *DKK*2 haplotype 1 in their tumor adjacent tissue. All eight HCC cases had chromosomal deletions of band 4q2l, and six cases showed LOH for 4q22-25 (S2 Table). Interestingly, three of the cases were polysomic and one was disomic for chromosome 4 with loss of 4q21, as determined by dual-color FISH. An example is shown in Fig 5. Sequencing of the *DKK*2 gene in the tumor tissue of these cases indicated that only haplotype 1 was retained in the tumor tissue, regardless of the copy number of 4q21 signals. These results support the idea that, during HCC tumorigenesis, chromosome amplification occurs at the *DKK2* locus prior to LOH (Fig 6).

## Discussion

In this study, we have taken a genetic approach to investigate the LOH region of human chromosome 4 and its role in HCC oncogenesis. By scrutinizing the genetic variants in a 37.2 Mb region of common chromosomal loss that affects nearly 60% of the HCC cases, we have uncovered the tumor suppressor function of DKK2 in the liver. Additionally, our study provides new insights regarding LOH in HCC.

First, we have shown that DKK2 function was compromised in HCC by the removal of active *DKK*2 alleles. The Wnt signaling pathway plays an important role in liver cancer, and extensive studies have revealed that Wnt antagonists can be inactivated by epigenetic modification of the DKK coding genes [22, 26]. By way of contrast, our finding provides a new mechanism whereby *DKK*2 loses its function through selective retention of an inactive allele (Fig 6). Thus, our data supports that this principle is also applicable to hepatocarcinogenesis.

Secondly, re-sequencing the LOH region allowed us to discover functional variants associated with hepatocarcinogenesis. By detecting the differential distribution of haplotypes between blood, non-tumor tissue, and tumor tissue (Table 3), we were able to identify significant genetic changes in the chromosomal regions showing genomic instability. Selective retention of a functional allele, in theory, could also give rise to overexpression of an oncogene. Allelic imbalance in combination with DNA amplification has been detected in the HCC genome. Given the frequent and extensive genomic changes associated with HCC, other tumor suppressor genes might also be inactivated through a similar mechanism. For example, *UNC5C* is a known tumor suppressor gene [31, 32]. Within the 4q21-25 region, *UNC5C* displayed a nonrandom distribution of alleles in the HCC tumors when LOH has occurred. The functional significance of the *ZGFR1* gene showing LOH is currently unknown.

Thirdly, by taking a comprehensive approach on a focused region, our analysis revealed that there was hyper-recombination in the promoter region of the *DKK*2 sequence (Table 3). We identified more than two haplotypes in the adjacent non-tumor liver tissues, yet most HCC cases retained haplotype 1 in the tumor tissues. Myers *et al*. (2008) reported that two DNA motifs are associated with recombination hot spots: the 7-mer CCTCCCT and the 13-mer CCNCCNTNNCCNC; these are clustered in breakpoint regions and act as a driver of genome instability [37]. We scanned the *DKK*2–1.5 kb to +1 kb region and found two CCTCCCT motifs in the *DKK*2 exon 1 sequence at +640 to +646 and +644 to +650 (S1 Fig). Furthermore, we searched ReDB (http://www.bioinf.seu.edu.cn/ReDatabase/), a recombination rate database to investigate the *DKK*2 locus. Interestingly, the recombination rate of the *DKK*2–437 to -4,276 promoter region was dramatically increased from average of 0.02% to 14.73% (S3 Table). Thus, the results of the sequence analysis support the scheme shown in Fig 6. As the cell proliferation rate is elevated in the pre-cancerous tissues, DNA breakage is likely to occur near the recombination hotspots in the *DKK*2 promoter region and this will lead to loss of *DKK*2 alleles. At the same time, those cells with low transcriptional activity of the *DKK*2 haplotype 1 allele are selected for clonal amplification during tumorigenesis.

Finally, our genetic and functional data confirms that DKK2 functions as a tumor suppressor in the liver. The results from the functional analysis using cultured liver cancer cell support the hypothesis that DKK2 acts through the canonical Wnt pathway and antagonize the cell proliferation elicited by the Wnt3a ligand (Fig 4). While this study was in progress, others studying different cancer types have reported that *DKK2* functions as a tumor suppressor gene [38–40]. Of particular relevance to liver cancer, Maass *et al*. (2015) recently published that a *Dkk2* deletion in mice is associated with liver carcinogenesis and enrichment of stem cell properties [41]. Thus, DKK2 might work through both Wnt-dependent and independent mechanisms during hepatocarcinogenesis. Considering the role of DKK2 in HCC oncogenesis, genes affected by DKK2 modulation could possibly serve as biomarkers in epidemiological studies. Additional work is warranted to address the implications of these findings with respect to disease classification and clinical management.

## Materials and Methods

### Ethics statement

The study was approved by the Research Ethics Committee of National Health Research Institutes (Permit Number: EC1030201-E) and informed consent was obtained from each participant. Human subjects were recruited from the Koo Foundation Sun Yat-Sen Cancer Center and Chang Gung Memorial Hospital. These human specimens were collected under informed consent in accordance with the recommendations of Research Ethics Committee of National Health Research Institutes.

### Specimen preparation

Genomic DNA and total RNA were isolated using the single-step method [42] from tumor tissues of the HCC patients as well as from their adjacent non-tumor tissues that appeared normal.

### Sequencing reactions and data analysis

Primers specifically targeting each genomic fragment were designed using Primer3. Primer sequence information on the 2,293 amplicons is available on request. PCR was initiated at 95°C for 10 minutes, followed by 45 cycles of 95°C for 30 seconds, annealing at various temperatures as appropriate to the primer pair for 30 seconds, and extension at 72°C for 45 seconds. The final step was at 72°C for 3 minutes. The optimal annealing temperature for each pair of primer was pre-tested. The PCR products were treated with exonuclease I in order to remove unreacted primers. DNA sequencing reactions were performed using Dye-terminator (Applied Biosystems Inc., Foster, CA) and the same primers were used for the PCR amplification. The products were separated by electrophoresis on an automated ABI 3700 PRISM DNA sequencer to determine the sequence of amplified fragments. The results were analyzed using Phrap-Phred and PolyPhred (ver. 10) software [43]. Heterozygous variations were identified by the presence of double peaks at single nucleotide positions.

### Haplotyping of the *DKK*2 gene

The forward primer 5'-TTTGCTTGGAAAGTCTCGC-3' and the reverse primer 5'-AGGGGTGGGAATGCAAAG-3' were used for PCR amplification of the -1,135 to +667 genomic region of the *DKK*2 gene. The PCR products were subjected to TA cloning using the pGEM-T vector (Promega). After transformation, 96 colonies were individually selected for direct sequencing.

### Transcriptional activity assay

A DNA fragment, -1,135 to +667 of the *DKK*2 gene, was amplified using genomic DNA from each of the HCC cases with different haplotypes. Sequence of the PCR product was verified before cloning into the pGL3 vector. In total, 4 μg of pGL3-*DKK*2 promoter plasmid DNA and 0.8 μg of pcDNA3.1-His-LacZ plasmid DNA were co-transfected into HuH-7 cells. After 48 hours, the cells were lyzed and the luciferase activity was detected by LucLite Kit (Packard BioScience) following the manufacture’s instruction. To report the relative activity, the measured luciferase activity was normalized against the activity of β-galactosidase activity, which served as a transfection control.

### Quantitative analysis of *DKK2* expression

Relative *DKK2* expression levels between the tumor (T) tissues and the non-tumor (N) counterpart were determined using RT-qPCR. Total RNA from 30 pairs of HCC samples were reverse-transcribed to cDNA using SuperScriptII (Invitrogen) according to the manufacturer's instructions. Subsequent qPCR reactions for *DKK2* and β-actin were performed in triplicates on ABI StepOne real-time PCR system, using KAPA SYBR FAST ABI Prism 2X qPCR Master Mix (Kapa Biosystems). The sequences of the primers used for RT-qPCR were as follows: for *DKK2*, 5’- GCAATAATGGCATCTGTATC (forward) and 5’- GTCTGATGATCGTAGGCAG (reverse) and for β-actin, 5’- ATCCGCAAAGACCTGTAC (forward) and 5’- GGAGGAGCAATGATCTTG (reverse). All samples were analyzed and normalized with expression level of the internal control gene, β-actin. Relative quantification of fold-change was performed, comparing △CT of tumor tissues and △CT of tumor adjacent tissues.

### TOPflash assay

For the TOPflash assay [35], 2 μg of TCF reporter plasmid DNA and 0.5 μg of pcDNA3.1-His-LacZ plasmid DNA were co-transfected into HuH-7 cells. After 24 hours, the cells were starved with DMEM medium containing 0.1% FBS for another 24 hours. Then, the cells were cultured for 48 hours with medium that contained Wnt3a and/or DKK2 recombinant protein (ng/ml) (Peprotech). The TOPflash activity was measured by luciferase activity using the Dual-Luciferase Reporter Assay Kit (Promega). The data was normalized against β-galactosidase activity.

### Cell proliferation assay

HuH-7 cells were plated in the 24 well plates (2x10^4^ cells per well) for 24 hours before the cells underwent serum starvation. After 24 hours, the cells were cultured with DMEM medium containing Wnt3a and/or DKK2 recombinant protein (ng/ml) for 48 hours. The cell proliferation assay was performed using alamarBlue cell viability reagent (Thermo Scientific) according to the user manual.

### Western blot

HuH-7 cells were serum-starved and stimulated with Wnt3a and/or DKK2 recombinant protein, as described above. But, after 6 hours, the nucleus and cytoplasm were separated using the ProteoJET Cytoplasmic and Nuclear Protein Extraction Kit (Fermentas). Protein samples, loaded with 20 μg per lane, were separated by electrophoresis on a 10% SDS-PAGE gel and transferred onto a membrane. Then, the membrane was probed with primary antibodies at optimal dilutions, followed by secondary antibody detection. The primary antibodies used for the current study were anti-β-catenin (Cell Signaling) and anti-GAPDH (Novus Biologicals) and anti-Histone H3 (Cell Signaling).

### Fluorescence *in situ* hybridization

Touch slide preparations, probe preparations and fluorescence *in situ* hybridization were performed according to published protocols [44]. In brief, a biotin-labeled 964 a_2 YAC probe specific to chromosome band 4q2l was cohybridized with a digoxigenin-labeled centromeric probe for chromosome 4. Signal detection was accomplished using avidin-FITC and rhodamine antidigoxigenin. Nuclear counterstaining was carried out using 0.1 μg/ml DAPI in antifade solution.

### Statistical analysis

To confirm significance of the data obtained from *in vivo* studies, Kruskal-Wallis *H* test was implemented to determine if the clusters were significantly different. After significance was established, Mann-Whitney *U* tests were used to identify which cluster exhibited the greatest significance. For *in vitro* data, variance pre-test was analyzed using the F test of equality of variances. Once the data sets were determined to show homoscedasticity, Student's t test was performed to test the significance of the differences between the sample conditions. To verify dose-dependence of cell proliferation rate, ANOVA for regression analysis was used.

## Supporting Information

S1 Fig The motif of the recombination hot spot in the DKK2–1.5 kb to +1 kb region. There are two CCTCCCT motifs in the DKK2 exon1 at +640 to +646 and +644 to +650. The sequences that are labeled in red represent the CCTCCCT motif and the black underlines indicate TCCCT motif 1 and CCTCCCT motif 2.
(TIF)

S1 Table Significant variations within human chromosome 4q21-25. (DOCX)

S2 Table Cytogenetic changes in eight HCC cases that were heterozygous for DKK2 haplotype 1 in their tumor adjacent tissue. (DOCX)

S3 Table The recombination rate in the DKK2 region plus 10 kb promoter region. (DOCX)

## References

[1] Jemal A, Siegel R, Ward E, Murray T, Xu J, Thun MJ. Cancer statistics, 2007. CA: a cancer journal for clinicians. 2007;57(1):43–66. Epub 2007/01/24. . 17237035 10.3322/canjclin.57.1.43

[2] Parkin DM. Global cancer statistics in the year 2000. The Lancet Oncology. 2001;2(9):533–43. Epub 2002/03/22. 10.1016/s1470-2045(01)00486-7 . 11905707

[3] El-Serag HB, Davila JA, Petersen NJ, McGlynn KA. The continuing increase in the incidence of hepatocellular carcinoma in the United States: an update. Annals of internal medicine. 2003;139(10):817–23. Epub 2003/11/19. . 14623619 10.7326/0003-4819-139-10-200311180-00009

[4] Herath NI, Leggett BA, MacDonald GA. Review of genetic and epigenetic alterations in hepatocarcinogenesis. Journal of gastroenterology and hepatology. 2006;21(1 Pt 1):15–21. Epub 2006/05/19. 10.1111/j.1440-1746.2005.04043.x . 16706806

[5] Balsara BR, Pei J, De Rienzo A, Simon D, Tosolini A, Lu YY, et al Human hepatocellular carcinoma is characterized by a highly consistent pattern of genomic imbalances, including frequent loss of 16q23.1–24.1. Genes, chromosomes & cancer. 2001;30(3):245–53. Epub 2001/02/15. . 11170281 10.1002/1098-2264(2000)9999:9999<::aid-gcc1083>3.0.co;2-m

[6] Hashimoto K, Mori N, Tamesa T, Okada T, Kawauchi S, Oga A, et al Analysis of DNA copy number aberrations in hepatitis C virus-associated hepatocellular carcinomas by conventional CGH and array CGH. Modern pathology: an official journal of the United States and Canadian Academy of Pathology, Inc. 2004;17(6):617–22. Epub 2004/05/11. 10.1038/modpathol.3800107 . 15133472

[7] Kawai H, Suda T, Aoyagi Y, Isokawa O, Mita Y, Waguri N, et al Quantitative evaluation of genomic instability as a possible predictor for development of hepatocellular carcinoma: comparison of loss of heterozygosity and replication error. Hepatology (Baltimore, Md). 2000;31(6):1246–50. Epub 2000/05/29. 10.1053/jhep.2000.7298 . 10827149

[8] Kusano N, Okita K, Shirahashi H, Harada T, Shiraishi K, Oga A, et al Chromosomal imbalances detected by comparative genomic hybridization are associated with outcome of patients with hepatocellular carcinoma. Cancer. 2002;94(3):746–51. Epub 2002/02/22. . 11857308 10.1002/cncr.10254

[9] Okabe H, Ikai I, Matsuo K, Satoh S, Momoi H, Kamikawa T, et al Comprehensive allelotype study of hepatocellular carcinoma: potential differences in pathways to hepatocellular carcinoma between hepatitis B virus-positive and -negative tumors. Hepatology (Baltimore, Md). 2000;31(5):1073–9. Epub 2000/05/05. 10.1053/he.2000.6409 . 10796882

[10] Wong N, Lai P, Pang E, Fung LF, Sheng Z, Wong V, et al Genomic aberrations in human hepatocellular carcinomas of differing etiologies. Clinical cancer research: an official journal of the American Association for Cancer Research. 2000;6(10):4000–9. Epub 2000/10/29. . 11051249

[11] Farazi PA, DePinho RA. Hepatocellular carcinoma pathogenesis: from genes to environment. Nature reviews Cancer. 2006;6(9):674–87. Epub 2006/08/25. 10.1038/nrc1934 . 16929323

[12] Nelson WJ, Nusse R. Convergence of Wnt, beta-catenin, and cadherin pathways. Science (New York, NY). 2004;303(5663):1483–7. Epub 2004/03/06. 10.1126/science.1094291 ; PubMed Central PMCID: PMCPmc3372896. 15001769 PMC3372896

[13] Edamoto Y, Hara A, Biernat W, Terracciano L, Cathomas G, Riehle HM, et al Alterations of RB1, p53 and Wnt pathways in hepatocellular carcinomas associated with hepatitis C, hepatitis B and alcoholic liver cirrhosis. International journal of cancer Journal international du cancer. 2003;106(3):334–41. Epub 2003/07/08. 10.1002/ijc.11254 . 12845670

[14] Ishizaki Y, Ikeda S, Fujimori M, Shimizu Y, Kurihara T, Itamoto T, et al Immunohistochemical analysis and mutational analyses of beta-catenin, Axin family and APC genes in hepatocellular carcinomas. International journal of oncology. 2004;24(5):1077–83. Epub 2004/04/07. . 15067328

[15] An FQ, Matsuda M, Fujii H, Tang RF, Amemiya H, Dai YM, et al Tumor heterogeneity in small hepatocellular carcinoma: analysis of tumor cell proliferation, expression and mutation of p53 AND beta-catenin. International journal of cancer Journal international du cancer. 2001;93(4):468–74. Epub 2001/07/31. . 11477549 10.1002/ijc.1367

[16] Peng SY, Chen WJ, Lai PL, Jeng YM, Sheu JC, Hsu HC. High alpha-fetoprotein level correlates with high stage, early recurrence and poor prognosis of hepatocellular carcinoma: significance of hepatitis virus infection, age, p53 and beta-catenin mutations. International journal of cancer Journal international du cancer. 2004;112(1):44–50. Epub 2004/08/12. 10.1002/ijc.20279 . 15305374

[17] Thorgeirsson SS, Grisham JW. Molecular pathogenesis of human hepatocellular carcinoma. Nature genetics. 2002;31(4):339–46. Epub 2002/08/01. 10.1038/ng0802-339 . 12149612

[18] Hsu HC, Jeng YM, Mao TL, Chu JS, Lai PL, Peng SY. Beta-catenin mutations are associated with a subset of low-stage hepatocellular carcinoma negative for hepatitis B virus and with favorable prognosis. The American journal of pathology. 2000;157(3):763–70. Epub 2000/09/12. ; PubMed Central PMCID: PMCPmc1885685. 10980116 10.1016/s0002-9440(10)64590-7 PMC1885685

[19] Huang H, Fujii H, Sankila A, Mahler-Araujo BM, Matsuda M, Cathomas G, et al Beta-catenin mutations are frequent in human hepatocellular carcinomas associated with hepatitis C virus infection. The American journal of pathology. 1999;155(6):1795–801. Epub 1999/12/14. ; PubMed Central PMCID: PMCPmc1866943. 10595907 10.1016/s0002-9440(10)65496-x PMC1866943

[20] Gross-Goupil M, Riou P, Emile JF, Saffroy R, Azoulay D, Lacherade I, et al Analysis of chromosomal instability in pulmonary or liver metastases and matched primary hepatocellular carcinoma after orthotopic liver transplantation. International journal of cancer Journal international du cancer. 2003;104(6):745–51. Epub 2003/03/18. 10.1002/ijc.11017 . 12640682

[21] Nhieu JT, Renard CA, Wei Y, Cherqui D, Zafrani ES, Buendia MA. Nuclear accumulation of mutated beta-catenin in hepatocellular carcinoma is associated with increased cell proliferation. The American journal of pathology. 1999;155(3):703–10. Epub 1999/09/17. ; PubMed Central PMCID: PMCPmc1866892. 10487827 10.1016/s0002-9440(10)65168-1 PMC1866892

[22] Fatima S, Lee NP, Luk JM. Dickkopfs and Wnt/beta-catenin signalling in liver cancer. World journal of clinical oncology. 2011;2(8):311–25. Epub 2011/08/31. 10.5306/wjco.v2.i8.311 ; PubMed Central PMCID: PMCPmc3163259. 21876852 PMC3163259

[23] Glinka A, Wu W, Delius H, Monaghan AP, Blumenstock C, Niehrs C. Dickkopf-1 is a member of a new family of secreted proteins and functions in head induction. Nature. 1998;391(6665):357–62. Epub 1998/02/05. 10.1038/34848 . 9450748

[24] Bafico A, Liu G, Yaniv A, Gazit A, Aaronson SA. Novel mechanism of Wnt signalling inhibition mediated by Dickkopf-1 interaction with LRP6/Arrow. Nature cell biology. 2001;3(7):683–6. Epub 2001/07/04. 10.1038/35083081 . 11433302

[25] Mao B, Wu W, Li Y, Hoppe D, Stannek P, Glinka A, et al LDL-receptor-related protein 6 is a receptor for Dickkopf proteins. Nature. 2001;411(6835):321–5. Epub 2001/05/18. 10.1038/35077108 . 11357136

[26] Sun X, He Y, Huang C, Ma TT, Li J. Distinctive microRNA signature associated of neoplasms with the Wnt/beta-catenin signaling pathway. Cellular signalling. 2013;25(12):2805–11. Epub 2013/09/18. 10.1016/j.cellsig.2013.09.006 . 24041653

[27] Li SP, Wang HY, Li JQ, Zhang CQ, Feng QS, Huang P, et al Genome-wide analyses on loss of heterozygosity in hepatocellular carcinoma in Southern China. Journal of hepatology. 2001;34(6):840–9. Epub 2001/07/14. . 11451167 10.1016/s0168-8278(01)00047-2

[28] Maggioni M, Coggi G, Cassani B, Bianchi P, Romagnoli S, Mandelli A, et al Molecular changes in hepatocellular dysplastic nodules on microdissected liver biopsies. Hepatology (Baltimore, Md). 2000;32(5):942–6. Epub 2000/10/26. 10.1053/jhep.2000.18425 . 11050043

[29] Yeh SH, Chen PJ, Shau WY, Chen YW, Lee PH, Chen JT, et al Chromosomal allelic imbalance evolving from liver cirrhosis to hepatocellular carcinoma. Gastroenterology. 2001;121(3):699–709. Epub 2001/08/28. . 11522754 10.1053/gast.2001.27211

[30] Knudson AG Jr. Mutation and cancer: statistical study of retinoblastoma. Proceedings of the National Academy of Sciences of the United States of America. 1971;68(4):820–3. Epub 1971/04/01. ; PubMed Central PMCID: PMCPmc389051. 5279523 10.1073/pnas.68.4.820 PMC389051

[31] Bernet A, Mazelin L, Coissieux MM, Gadot N, Ackerman SL, Scoazec JY, et al Inactivation of the UNC5C Netrin-1 receptor is associated with tumor progression in colorectal malignancies. Gastroenterology. 2007;133(6):1840–8. Epub 2007/10/31. 10.1053/j.gastro.2007.08.009 ; PubMed Central PMCID: PMCPmc2211510. 17967459 PMC2211510

[32] Shin SK, Nagasaka T, Jung BH, Matsubara N, Kim WH, Carethers JM, et al Epigenetic and genetic alterations in Netrin-1 receptors UNC5C and DCC in human colon cancer. Gastroenterology. 2007;133(6):1849–57. Epub 2007/9/5. 10.1053/j.gastro.2007.08.074 . 18054557 PMC4139066

[33] Niehrs C. Function and biological roles of the Dickkopf family of Wnt modulators. Oncogene. 2006;25(57):7469–81. . 17143291 10.1038/sj.onc.1210054

[34] Bader JS. The relative power of SNPs and haplotype as genetic markers for association tests. Pharmacogenomics. 2001;2(1):11–24. Epub 2001/03/22. 10.1517/14622416.2.1.11 . 11258193

[35] Ishitani T, Ninomiya-Tsuji J, Nagai S, Nishita M, Meneghini M, Barker N, et al The TAK1-NLK-MAPK-related pathway antagonizes signalling between beta-catenin and transcription factor TCF. Nature. 1999;399(6738):798–802. Epub 1999/07/03. 10.1038/21674 . 10391247

[36] Klaus A, Birchmeier W. Wnt signalling and its impact on development and cancer. Nature reviews Cancer. 2008;8(5):387–98. Epub 2008/04/25. 10.1038/nrc2389 . 18432252

[37] Myers S, Freeman C, Auton A, Donnelly P, McVean G. A common sequence motif associated with recombination hot spots and genome instability in humans. Nature genetics. 2008;40(9):1124–9. Epub 2009/01/24. 10.1038/ng.213 . 19165926

[38] Hirata H, Hinoda Y, Nakajima K, Kawamoto K, Kikuno N, Kawakami K, et al Wnt antagonist gene DKK2 is epigenetically silenced and inhibits renal cancer progression through apoptotic and cell cycle pathways. Clinical cancer research: an official journal of the American Association for Cancer Research. 2009;15(18):5678–87. Epub 2009/09/17. 10.1158/1078-0432.ccr-09-0558 . 19755393

[39] Kawakita A, Yanamoto S, Yamada S, Naruse T, Takahashi H, Kawasaki G, et al MicroRNA-21 Promotes Oral Cancer Invasion via the Wnt/beta-Catenin Pathway by Targeting DKK2. Pathology oncology research: POR. 2014;20(2):253–61. Epub 2013/09/04. 10.1007/s12253-013-9689-y . 23999978

[40] Zhu J, Zhang S, Gu L, Di W. Epigenetic silencing of DKK2 and Wnt signal pathway components in human ovarian carcinoma. Carcinogenesis. 2012;33(12):2334–43. Epub 2012/09/12. 10.1093/carcin/bgs278 . 22964660

[41] Maass T, Marquardt J, Lee JS, Krupp M, Scholz-Kreisel P, Mogler C, et al Increased liver carcinogenesis and enrichment of stem cell properties in livers of Dickkopf 2 (Dkk2) deleted mice. Oncotarget. 2015 Epub 2015/04/01. . 25826080 10.18632/oncotarget.3293 PMC5045365

[42] Chomczynski P. A reagent for the single-step simultaneous isolation of RNA, DNA and proteins from cell and tissue samples. BioTechniques. 1993;15(3):532–4, 6–7. Epub 1993/09/01. . 7692896

[43] Ewing B, Hillier L, Wendl MC, Green P. Base-calling of automated sequencer traces using phred. I. Accuracy assessment. Genome research. 1998;8(3):175–85. Epub 1998/05/16. . 9521921 10.1101/gr.8.3.175

[44] Huang SF, Hsu HC, Chen JC, Chie WC, Lai P. Tetrasomy 6 and 6q14 deletion are associated with better survival in hepatocellular carcinomas. a fluorescence in situ hybridization study of 77 cases. Cancer genetics and cytogenetics. 2003;144(1):23–30. Epub 2003/06/18. . 12810252 10.1016/s0165-4608(02)00860-9

