# Supplementary figures and images for: Selective Retention of an Inactive Allele of the DKK2 Tumor Suppressor Gene in Hepatocellular Carcinoma
Source: PLoS Genet. 2016 May 20;12(5):e1006051. doi: 10.1371/journal.pgen.1006051 (PMC4874628; doi:10.1371/journal.pgen.1006051)

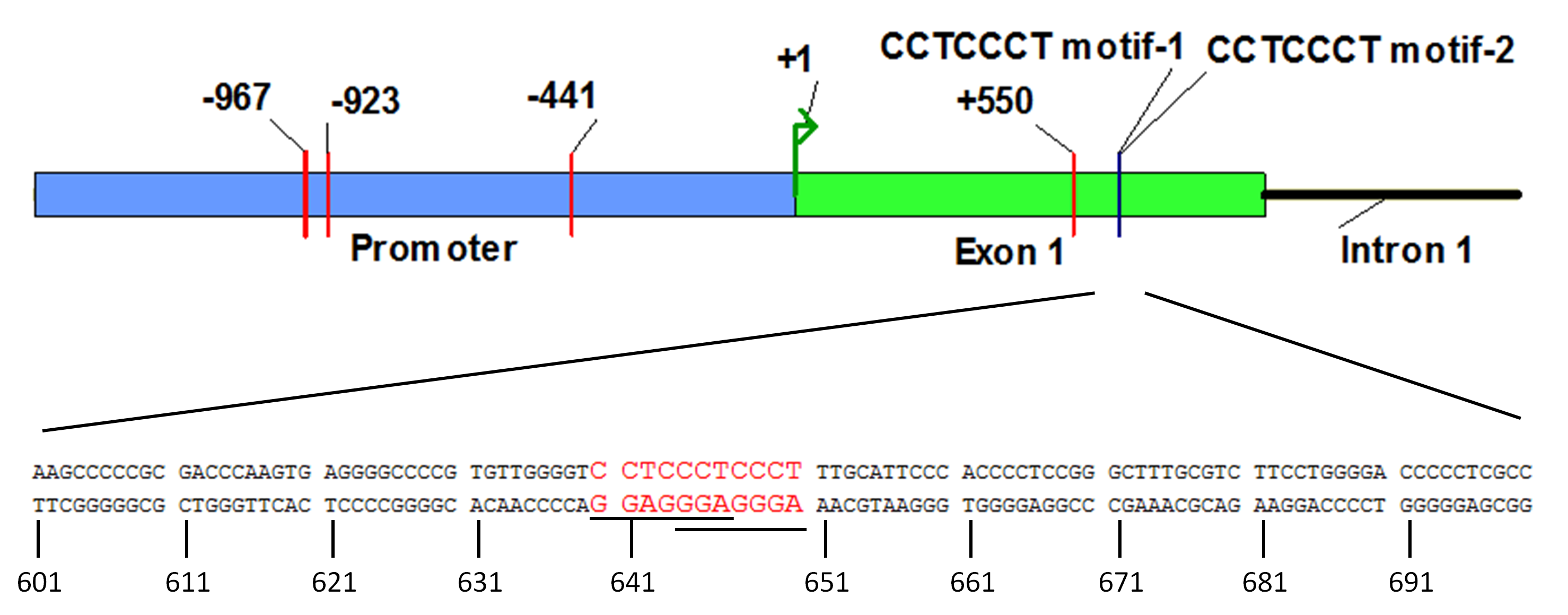

Supplement: S1 Fig — There are two CCTCCCT motifs in the DKK2 exon1 at +640 to +646 and +644 to +650. The sequences that are labeled in red represent the CCTCCCT motif and the black underlines indicate TCCCT motif 1 and CCTCCCT motif 2. (TIF) [file pgen.1006051.s001.tif]
