# Supplementary material for: Selective Retention of an Inactive Allele of the DKK2 Tumor Suppressor Gene in Hepatocellular Carcinoma
Source: PLoS Genet. 2016 May 20;12(5):e1006051. doi: 10.1371/journal.pgen.1006051 (PMC4874628; doi:10.1371/journal.pgen.1006051)
Supplement: S1 Table — (DOCX) [file pgen.1006051.s002.docx]

Supplementary Table S1. Significant variations within human chromosome 4q21-25.

| Chrom | ChromStart | ChromEnd | Gene Symbol | Type | Ref | Var | pValue | HGbuild |
| --- | --- | --- | --- | --- | --- | --- | --- | --- |
| chr4 | 76981912 | 76981912 | CDKL2 | Substitution | T | C | 0.03300 | 33 |
| chr4 | 77343555 | 77343555 | FLJ10498 | Substitution | A | G | 0.02900 | 33 |
| chr4 | 77497440 | 77497440 | NUP54 | Substitution | T | C | 0.01100 | 33 |
| chr4 | 77540466 | 77540466 | SCARB2 | Substitution | C | G | 0.02100 | 33 |
| chr4 | 77555522 | 77555522 | SCARB2 | Substitution | A | G | 0.01800 | 33 |
| chr4 | 77555834 | 77555834 | SCARB2 | Substitution | A | G | 0.02100 | 33 |
| chr4 | 77814498 | 77814498 | ShrmL | Substitution | G | A | 0.03800 | 33 |
| chr4 | 79288978 | 79288978 | MRPL1 | Substitution | C | T | 0.02000 | 33 |
| chr4 | 79445276 | 79445276 | FRAS1 | Substitution | G | A | 0.00200 | 33 |
| chr4 | 79445678 | 79445678 | FRAS1 | Substitution | A | C | 0.00200 | 33 |
| chr4 | 79445679 | 79445679 | FRAS1 | Substitution | C | A | 0.00200 | 33 |
| chr4 | 79445722 | 79445722 | FRAS1 | Substitution | A | G | 0.00200 | 33 |
| chr4 | 79646554 | 79646554 | FLJ14927 | Substitution | C | T | 0.03300 | 33 |
| chr4 | 80787257 | 80787257 | GK2 | Substitution | C | T | 0.00100 | 33 |
| chr4 | 80787308 | 80787308 | GK2 | Substitution | A | T | 0.00900 | 33 |
| chr4 | 80787531 | 80787531 | GK2 | Substitution | A | C | 0.05000 | 33 |
| chr4 | 80787593 | 80787593 | GK2 | Substitution | C | T | 0.00900 | 33 |
| chr4 | 80787836 | 80787836 | GK2 | Substitution | C | T | 0.02700 | 33 |
| chr4 | 84177920 | 84177920 | SCD4 | Substitution | T | C | 0.00500 | 33 |
| chr4 | 84296656 | 84296656 | THAP9 | Substitution | G | T | 0.00900 | 33 |
| chr4 | 84296656 | 84296656 | THAP9 | Substitution | G | T | 0.02800 | 33 |
| chr4 | 84307712 | 84307712 | DKFZp686L1814 | Substitution | C | T | 0.02900 | 33 |
| chr4 | 84315643 | 84315643 | DKFZp686L1814 | Substitution | A | TC | 0.00400 | 33 |
| chr4 | 84315644 | 84315644 | DKFZp686L1814 | Substitution | T | C | 0.00100 | 33 |
| chr4 | 84319356 | 84319356 | DKFZp686L1814 | Substitution | T | C | 0.01100 | 33 |
| chr4 | 84363747 | 84363747 | DKFZp686L1814 | Substitution | G | C | 0.01700 | 33 |
| chr4 | 84840739 | 84840739 | FLJ13614 | Substitution | T | A | 0.01600 | 33 |
| chr4 | 84916395 | 84916395 | MGC11324 | Substitution | A | G | 0.04000 | 33 |
| chr4 | 84916422 | 84916422 | MGC11324 | Substitution | C | G | 0.02700 | 33 |
| chr4 | 84924249 | 84924249 | MGC11324 | Substitution | A | C | 0.00200 | 33 |
| chr4 | 88684510 | 88684510 | SCDR9 | Substitution | G | C | 0.00500 | 33 |
| chr4 | 90034974 | 90034974 | HERC3 | Substitution | C | T | 0.01500 | 33 |
| chr4 | 90066618 | 90066618 | HERC3 | Substitution | C | T | 0.04300 | 33 |
| chr4 | 90107679 | 90107679 | FAM13A1 | Substitution | T | C | 0.04700 | 33 |
| chr4 | 90111869 | 90111869 | FAM13A1 | Substitution | C | T | 0.04400 | 33 |
| chr4 | 90202596 | 90202596 | FAM13A1 | Substitution | G | T | 0.00200 | 33 |
| chr4 | 90626707 | 90626707 | LOC285513 | Substitution | G | A | 0.01400 | 33 |
| chr4 | 90626711 | 90626711 | LOC285513 | Substitution | A | G | 0.01400 | 33 |
| chr4 | 90627144 | 90627144 | LOC285513 | Substitution | C | T | 0.00300 | 33 |
| chr4 | 90627670 | 90627670 | LOC285513 | Substitution | C | T | 0.00300 | 33 |
| chr4 | 96496644 | 96496644 | UNC5C | Substitution | T | A | 0.02000 | 33 |
| chr4 | 96516760 | 96516760 | UNC5C | Substitution | G | A | 0.00053 | 33 |
| chr4 | 96650354 | 96650354 | UNC5C | Substitution | C | T | 0.03000 | 33 |
| chr4 | 99351862 | 99351862 | MGC46496 | Substitution | G | A | 0.05000 | 33 |
| chr4 | 99351901 | 99351901 | MGC46496 | Substitution | T | C | 0.05000 | 33 |
| chr4 | 100451306 | 100451306 | ADH5 | Substitution | C | A | 0.00000 | 33 |
| chr4 | 100451319 | 100451319 | ADH5 | Substitution | A | G | 0.00000 | 33 |
| chr4 | 100724932 | 100724932 | ADH1C | Substitution | A | G | 0.03600 | 33 |
| chr4 | 100926852 | 100926852 | MGC27034 | Substitution | T | G | 0.00000 | 33 |
| chr4 | 100926858 | 100926858 | MGC27034 | Substitution | T | A | 0.00500 | 33 |
| chr4 | 100926859 | 100926859 | MGC27034 | Substitution | A | T | 0.00500 | 33 |
| chr4 | 100926861 | 100926861 | MGC27034 | Substitution | T | C | 0.00500 | 33 |
| chr4 | 100926862 | 100926862 | MGC27034 | Substitution | T | G | 0.02700 | 33 |
| chr4 | 100926866 | 100926866 | MGC27034 | Substitution | T | C | 0.00000 | 33 |
| chr4 | 101565915 | 101565915 | LOC115265 | Substitution | T | C | 0.00700 | 33 |
| chr4 | 101565927 | 101565927 | LOC115265 | Substitution | G | T | 0.00800 | 33 |
| chr4 | 101566787 | 101566787 | LOC115265 | Substitution | G | A | 0.00400 | 33 |
| chr4 | 101566846 | 101566846 | LOC115265 | Substitution | C | T | 0.01900 | 33 |
| chr4 | 101566909 | 101566909 | LOC115265 | Substitution | T | C | 0.01900 | 33 |
| chr4 | 101566921 | 101566921 | LOC115265 | Substitution | G | T | 0.01900 | 33 |
| chr4 | 107033896 | 107033896 | FLJ13273 | Substitution | T | G | 0.00647 | 33 |
| chr4 | 108350110 | 108350110 | DKK2 | Substitution | T | C | 0.00001 | 33 |
| chr4 | 108351090 | 108351090 | DKK2 | Substitution | T | G | 0.00249 | 33 |
| chr4 | 108351581 | 108351581 | DKK2 | Substitution | A | C | 0.00912 | 33 |
| chr4 | 108351625 | 108351625 | DKK2 | Substitution | T | A | 0.00912 | 33 |
| chr4 | 109250591 | 109250591 | LOC113612 | Substitution | A | G | 0.01000 | 33 |
| chr4 | 109920355 | 109920355 | FLJ37673 | Substitution | A | C | 0.03300 | 33 |
| chr4 | 110121462 | 110121463 | AGXT2L1 | Insertion | NNN | TGA | 0.00664 | 33 |
| chr4 | 110121538 | 110121538 | AGXT2L1 | Substitution | T | C | 0.01000 | 33 |
| chr4 | 110121756 | 110121756 | AGXT2L1 | Substitution | C | T | 0.00129 | 33 |
| chr4 | 110128414 | 110128414 | COL25A1 | Substitution | A | G | 0.02000 | 33 |
| chr4 | 110203795 | 110203795 | COL25A1 | Deletion | C | N | 0.01000 | 33 |
| chr4 | 110778143 | 110778143 | SEC24B | Substitution | T | C | 0.01000 | 33 |
| chr4 | 110845741 | 110845741 | SEC24B | Substitution | T | C | 0.03000 | 33 |
| chr4 | 110978850 | 110978850 | FLJ20647 | Substitution | G | A | 0.03000 | 33 |
| chr4 | 111002996 | 111002996 | CASP6 | Substitution | G | A | 0.00867 | 33 |
| chr4 | 111074710 | 111074710 | IF | Substitution | G | A | 0.00282 | 33 |
| chr4 | 111117404 | 111117405 | IF | Insertion | N | G | 0.01000 | 33 |
| chr4 | 111319143 | 111319143 | EGF | Substitution | G | A | 0.04000 | 33 |
| chr4 | 111326209 | 111326209 | EGF | Substitution | T | C | 0.02000 | 33 |
| chr4 | 111857345 | 111857345 | ENPEP | Substitution | T | G | 0.05000 | 33 |
| chr4 | 113569157 | 113569157 | FLJ39370 | Substitution | G | A | 0.04500 | 33 |
| chr4 | 113569161 | 113569161 | FLJ39370 | Substitution | C | G | 0.04500 | 33 |
| chr4 | 113569162 | 113569162 | FLJ39370 | Substitution | C | G | 0.04500 | 33 |
| chr4 | 113569163 | 113569163 | FLJ39370 | Substitution | T | A | 0.04500 | 33 |
| chr4 | 113569164 | 113569164 | FLJ39370 | Substitution | T | G | 0.04500 | 33 |
| chr4 | 113592089 | 113592090 | T2BP | Deletion | TC | NN | 0.00002 | 33 |
| chr4 | 113592091 | 113592092 | T2BP | Deletion | TT | NN | 0.00002 | 33 |
| chr4 | 113592094 | 113592095 | T2BP | Deletion | GT | NN | 0.00002 | 33 |
| chr4 | 113592280 | 113592280 | T2BP | Substitution | T | C | 0.00016 | 33 |
| chr4 | 113920277 | 113920277 | FLJ11331 | Substitution | T | C | 0.00077 | 33 |
| chr4 | 113933174 | 113933174 | FLJ11331 | Substitution | A | G | 0.03000 | 33 |
| chr4 | 113938370 | 113938370 | FLJ11331 | Substitution | A | C | 0.00170 | 33 |
| chr4 | 113942385 | 113942385 | LOC91431 | Substitution | G | T | 0.01000 | 33 |
| chr4 | 113947590 | 113947590 | FLJ11331 | Substitution | T | A | 0.01000 | 33 |
| chr4 | 113952474 | 113952474 | FLJ11331 | Substitution | A | G | 0.00891 | 33 |
| chr4 | 114027954 | 114027954 | HDCMA18P | Substitution | G | T | 0.00700 | 33 |
| chr4 | 114029458 | 114029458 | HDCMA18P | Substitution | C | A | 0.00800 | 33 |
| chr4 | 114037436 | 114037436 | HDCMA18P | Substitution | C | T | 0.00800 | 33 |
