# Supplementary material for: Selective Retention of an Inactive Allele of the DKK2 Tumor Suppressor Gene in Hepatocellular Carcinoma
Source: PLoS Genet. 2016 May 20;12(5):e1006051. doi: 10.1371/journal.pgen.1006051 (PMC4874628; doi:10.1371/journal.pgen.1006051)
Supplement: S2 Table — (DOCX) [file pgen.1006051.s003.docx]

Supplementary Table S2. Cytogenetic changes in eight HCC cases that were heterozygous for *DKK2* haplotype 1 in their tumor adjacent tissue

|  | chromosome 4 | 4q21 | | 4q22 | | 4q25 | | | *DKK2* |
| --- | --- | --- | --- | --- | --- | --- | --- | --- | --- |
|  | centromere probe | YAC probe | | 92.5M | 95.6M | 106.1M | 108.4M | 109.1M | haplotype 1 (TAGC) |
| sample ID | copy # | loss | 4q21/cen | D4S  414 | D4S  2433 | D4S  1570 | *DKK2* | D4S  2917 | tumor adjacent |
| CG-08 | polysomy | Y | 2/4 or 2/3 | ● | ○ | ● | ● | ／ | Y |
| CG-10 | monosomy (66%) | Y | 1/1 | ○ | ／ | ○ | ○ | ／ | Y |
| CG-11 | polysomy | Y | 3/5 or 2/5 | ● | ● | ○ | ● | ● | Y |
| CG-14 | polysomy | Y | 2/4 or 2/3 | ● | ● | ● | ● | ／ | Y |
| CG-17 | disomy | N | 2/2 | ○ | ／ | ● | ● | ／ | Y |
| CG-19 | monosomy (44%) | Y | 1/1 | ○ | ○ | ／ | ○ | ／ | Y |
| CG-20 | monosomy (60%) | Y | 1/1 | ／ | ● | ● | ○ | ● | Y |
| CG-22 | monosomy (60%) | Y | 1/1 | ／ | ○ | ／ | ○ | ● | Y |
| ● LOH ○ Non-LOH ／ Non-informative | | | |  |  |  |  |  |  |
