# Supplementary material for: Selective Retention of an Inactive Allele of the DKK2 Tumor Suppressor Gene in Hepatocellular Carcinoma
Source: PLoS Genet. 2016 May 20;12(5):e1006051. doi: 10.1371/journal.pgen.1006051 (PMC4874628; doi:10.1371/journal.pgen.1006051)
Supplement: S3 Table — (DOCX) [file pgen.1006051.s004.docx]

Supplementary Table S3. The recombination rate in the DKK2 region plus 10 kb promoter region.

| Chrom | ChromStart | ChromEnd | Ranking | Recombination Rate % | DKK2 Region |
| --- | --- | --- | --- | --- | --- |
| chr4 | 107842437 | 107849871 | 78.24% | 0.0601 | Exon2, Exon3, Exon4 |
| chr4 | 107849871 | 107853796 | 78.17% | 0.0604 | Intron1 |
| chr4 | 107853796 | 107861689 | 74.46% | 0.0791 | Intron1 |
| chr4 | 107861689 | 107862015 | 57.56% | 0.2021 | Intron1 |
| chr4 | 107862015 | 107883098 | 46.13% | 0.3452 | Intron1 |
| chr4 | 107883098 | 107884010 | 16.23% | 1.9371 | Intron1 |
| chr4 | 107884010 | 107888153 | 11.89% | 2.8844 | Intron1 |
| chr4 | 107888153 | 107888322 | 24.04% | 1.0956 | Intron1 |
| chr4 | 107888322 | 107890609 | 48.69% | 0.3068 | Intron1 |
| chr4 | 107890609 | 107892032 | 48.74% | 0.306 | Intron1 |
| chr4 | 107892032 | 107901832 | 48.76% | 0.3057 | Intron1 |
| chr4 | 107901832 | 107902236 | 55.96% | 0.2183 | Intron1 |
| chr4 | 107902236 | 107902285 | 63.12% | 0.1536 | Intron1 |
| chr4 | 107902285 | 107902442 | 72.61% | 0.0897 | Intron1 |
| chr4 | 107902442 | 107910185 | 86.53% | 0.0261 | Intron1 |
| chr4 | 107910185 | 107910744 | 87.11% | 0.0241 | Intron1 |
| chr4 | 107910744 | 107911443 | 87.47% | 0.0228 | Intron1 |
| chr4 | 107911443 | 107911895 | 87.77% | 0.0218 | Intron1 |
| chr4 | 107911895 | 107913107 | 88.12% | 0.0206 | Intron1 |
| chr4 | 107913107 | 107913621 | 88.45% | 0.0196 | Intron1 |
| chr4 | 107913621 | 107913996 | 88.70% | 0.0188 | Intron1 |
| chr4 | 107913996 | 107923574 | 88.99% | 0.0178 | Intron1 |
| chr4 | 107923574 | 107945079 | 89.01% | 0.0178 | Intron1 |
| chr4 | 107945079 | 107945561 | 89.00% | 0.0178 | Intron1 |
| chr4 | 107945561 | 107945637 | 89.00% | 0.0178 | Intron1 |
| chr4 | 107945637 | 107945881 | 89.00% | 0.0178 | Intron1 |
| chr4 | 107945881 | 107947287 | 89.00% | 0.0178 | Intron1 |
| chr4 | 107947287 | 107947533 | 89.00% | 0.0178 | Intron1 |
| chr4 | 107947533 | 107949788 | 88.99% | 0.0178 | Intron1 |
| chr4 | 107949788 | 107949916 | 88.96% | 0.0179 | Intron1 |
| chr4 | 107949916 | 107950671 | 88.98% | 0.0179 | Intron1 |
| chr4 | 107950671 | 107957133 | 88.97% | 0.0179 | Exon1 +321 to Intron1 +6782 |
| chr4 | 107957133 | 107957206 | 88.51% | 0.0194 | Exon1 +248 to Exon1 +321 |
| chr4 | 107957206 | 107957250 | 87.86% | 0.0215 | Exon1 +204 to Exon1 +248 |
| chr4 | 107957250 | 107957890 | 87.27% | 0.0235 | Promoter -437 to Exon1 +204 |
| chr4 | 107957890 | 107957933 | 14.30% | 2.2908 | Promoter -437 to Promoter -480 |
| chr4 | 107957933 | 107958252 | 1.50% | 15.4341 | Promoter -480 to Promoter -799 |
| chr4 | 107958252 | 107958624 | 1.17% | 17.5999 | Promoter -799 to Promoter -1171 |
| chr4 | 107958624 | 107958748 | 1.09% | 18.2143 | Promoter -1171 to Promoter -1295 |
| chr4 | 107958748 | 107959122 | 1.03% | 18.8378 | Promoter -1295 to Promoter -1669 |
| chr4 | 107959122 | 107959960 | 0.92% | 19.8201 | Promoter -1669 to Promoter -2507 |
| chr4 | 107959960 | 107961377 | 0.89% | 20.1437 | Promoter -2507 to Promoter -3924 |
| chr4 | 107961377 | 107961729 | 6.39% | 5.4896 | Promoter -3924 to Promoter -4276 |
| chr4 | 107961729 | 107962873 | 17.78% | 1.7035 | Promoter -4276 to Promoter -5420 |
| chr4 | 107962873 | 107963212 | 19.20% | 1.5281 | Promoter -5420 to Promoter -5759 |
| chr4 | 107963212 | 107963752 | 20.10% | 1.4293 | Promoter -5759 to Promoter -6299 |
| chr4 | 107963752 | 107964368 | 20.56% | 1.3839 | Promoter -6299 to Promoter -6915 |
| chr4 | 107964368 | 107967719 | 21.06% | 1.3373 | Promoter -6915 to Promoter -10266 |
